# Adaptation and implementation of a parenting curriculum in a refugee/immigrant community using a task-shifting approach: a study protocol

**DOI:** 10.1186/s12889-021-11148-2

**Published:** 2021-06-06

**Authors:** Daniel J. Whitaker, Shannon Self-Brown, Erin A. Weeks, Mary Helen O’Connor, Matthew Lyons, Cathleen Willging, Nae Hyung Lee, Jessica L. Kumar, Hannah Joseph, Dennis E. Reidy, Danielle Rivers, Nikita Rao

**Affiliations:** 1 Georgia State University, Atlanta, USA; 2 Pacific Institute for Research and Evaluation, Albuquerque, USA

## Abstract

**Background:**

Delivering evidence-based interventions to refugee and immigrant families is difficult for several reasons, including language and cultural issues, and access and trust issues that can lead to an unwillingness to engage with the typical intervention delivery systems. Adapting both the intervention and the delivery system for evidence-based interventions can make those interventions more appropriate and palatable for the targeted population, increasing uptake and effectiveness. This study focuses on the adaptation of the SafeCare© parenting model, and its delivery through either standard implementation methods via community-based organizations (CBO) and a task-shifted implementation in which members of the Afghans, Burmese, Congolese community will be trained to deliver SafeCare.

**Method:**

An adaptation team consisting of community members, members of CBO, and SafeCare experts will engage a structured process to adapt the SafeCare curriculum for each targeted community. Adaptations will focus on both the model and the delivery of it. Data collection of the adaptation process will focus on documenting adaptations and team member’s engagement and satisfaction with the process. SafeCare will be implemented in each community in two ways: standard implementation and task-shifted implementation. Standard implementation will be delivered by CBOs (*n* = 120), and task-shifted implementation will be delivered by community members (*n* = 120). All interventionists will be trained in a standard format, and will receive post-training support. Both implementation metrics and family outcomes will be assessed. Implementation metrics will include ongoing adaptations, delivery of services, fidelity, skill uptake by families, engagement/completion, and satisfaction with services. Family outcomes will include assessments at three time points (pre, post, and 6 months) of positive parenting, parent-child relationship, parenting stress, and child behavioral health.

**Discussion:**

The need for adapting of evidence-based programs and delivery methods for specific populations continues to be an important research question in implementation science. The goal of this study is to better understand an adaptation process and delivery method for three unique populations. We hope the study will inform other efforts to deliver health intervention to refugee communities and ultimately improve refugee health.

## Contributions to the literature

- There is a strong need for implementation of evidence-based mental and behavioral health interventions among refugee and immigrant populations, yet models for adapting and implementing those interventions have not be broadly tested.
- We will implement an adaptation process to adapt an evidence-based parenting model (SafeCare) for three refugee groups in a US-based refugee resettlement zone.
- We will test a standard implementation model against a task-shifted implementation model in which lay members of the community delivery the intervention.

## Introduction

According to the World Health Organization, the global population is more mobile than at any other point in history, with 1 billion migrants, 250 million international migrants, and at least 80 million people being forcibly displaced [90]. There is growing consensus that refugee and immigrant health is the public health crisis of this century [87]. Refugees face traumatic experiences that force migration, occur during migration, and occur even once they arrive in their host countries [63]. In fact, post-migratory stressors faced by refugees can be greater than pre-migratory stressors [86]. Those stressors can include poverty, social exclusion, discrimination and isolation, communication issues, violence and family conflict, lack of employment opportunities, poor working conditions, and dependency on public services. The number of migrants and the health problems they face have resulted in a significant strain on public health infrastructure in nations where refugees settle [98].

The physical health needs of refugees can be more evident than other issues, and targeted interventions focus on physical needs such as poor nutrition or infectious disease [38]. Mental health needs, though often overlooked, are equally important. The need for interventions focused on refugee mental health continues to grow [10, 73]. Though data are scarce on the prevalence of mental health problems among refugees, a 2018 study of nearly 2000 Syrian refugees found that 43% of the sample met diagnostic criteria for PTSD [27]. Both pre-migratory factors such as war trauma and post-migratory factors such as discrimination and non-permanent legal status are also associated with poor mental health [65]. Among refugee children, studies demonstrate very high rates of mental health issues with high rates of PTSD at 54% [15], depression at 30% [15], anxiety at 27% [11], and traumatic grief and behavioral problems at 24% [11]. Exposures to violence and other adverse experiences before, during, and after the forced migration experience have been identified through systematic review as a key risk factor for poor mental health outcomes among refugee children relocating to high income countries, while social support and stable settlement in the host community have been identified as key protective factors [40]. Minimizing post-migratory risk factors and bolstering protective factors are key in addressing the mental health needs of refugee children.

### Addressing toxic stress by promoting parenting

Chronic activation of the stress response system, or so called toxic stress, can produce negative physiological, psychological, and behavioral changes in children [82]. One strong protective mechanism for the impact of toxic stress is the presence of a safe, stable and nurturing relationship with an adult [82]. Children who experience stressors but have a nurturing adult in their lives are less likely to experience those stressors as toxic [46]. Positive parenting has been shown to buffer children from the negative biological impacts of stressors such as changes to allostatic load [14], pro-inflammatory signaling profiles [26], metabolic profiles [62], and hippocampal and amygdalar development [54]. Parenting programs can bolster parents’ ability to provide warm, supportive relationships and a safe environment [88], buffer children from the impact of toxic stress [83], and lead to positive social, emotional and behavioral development of children [70]. Parenting programs are a central strategy to prevent/address child maltreatment [8, 94] with several studies finding behaviorally-based parenting programs reduce child maltreatment rates [23, 24, 71, 74]. Parenting programs also improve parent mental health with meta-analyses showing that parenting programs are associated with reduced depression, stress, and anxiety [5]. A range of authoritative public health sources support positive parenting programs as a medium for promoting children’s mental and physical health including the CDC [21], WHO [97], and the Harvard Center on the Developing Child [69].

A primary question, then, in addressing the mental and behavioral health of refugee and immigrant children, is how to implement behaviorally-based and evidence-based parenting programs in refugee and immigrant populations. Two key challenges in this implementation are: (1) if and how to adapt the curriculum and, (2) whether the typical implementation delivery system is suited to deliver interventions to this population. Aarons and colleagues [2] describe the process of applying interventions to new populations and new service systems as issues of “scaling out,” and suggest adaptations must be considered when scaling out. Implementation among refugees may require unique adaptations to foster an effective scaling out of the intervention.

#### Curriculum adaptation

A first challenge in the delivery of an EBP to a refugee and immigrant population is how to adapt the intervention for the targeted populations. Although some data show that evidence-based parenting programs without adaptations can produce positive effects [22, 58] and that those programs transport well across countries [43], there is growing consensus for the need to consider cultural adaptations both for program effectiveness and to ensure acceptability and uptake by the targeted populations [9, 20, 50, 51, 60]. Cultural adaptations of standardized interventions have been found to be generally effective at producing desired outcomes [47] including adapted parenting interventions [9, 58, 72]. However, program engagement and uptake is generally poor and prevention programming is underutilized by members of racial and ethnic minorities including refugees [17]. There is limited work on adapted interventions for resettled refugee and immigrant populations [4, 39, 85], but several theoretical approaches have been proposed as part of the adaptation process. A minimal approach is to check the cultural relevance of the content by having members of the targeted population review materials [60]. More in-depth adaptations can be made by embedding the intervention into the cultural concepts and community [72]. Here we follow the Dynamic Adaptation Process [1] in identifying adaptations to both curriculum and implementation processes in adapting a parenting program for three different ethnic groups.

#### Implementation delivery system: the importance of task shifting

A second challenge in delivering EBPs to immigrants and refugees pertains to the implementation delivery system. Typically, delivery is handled by public service systems that fund private or non-profit community-based organizations for service delivery. Refugee and immigrants may be in a poor position to access such systems. They often do not speak English, and may be mistrustful of governmental systems [17], and may have cultural beliefs inconsistent with those espoused within U.S. service systems. Adaptations to how EBPs are delivered and who typically delivers them may be needed to reach refugee populations in the U.S.

The World Health Organization (WHO) suggests “beginning with the end in mind,” only considering solutions that might be candidates for scale-up and sustainability. One potential solution is to employ the concept of task-shifting. Task shifting involves “the rational redistribution of tasks among health workforce team” [96] and moving specific tasks from qualified and trained health workers to lay workers or community members without formal training. The goal is to make efficient use of the available work force and to meet the needs of the local population. Task shifting is used extensively in developing countries to address the shortage of mental health services and professionals [49]. Task shifting has been successfully applied training lay people in the treatment of depression [13, 49, 92], family psychoeducation for schizophrenia [75] and other psychotic disorders [25], as well as for anxiety and mood issues in Iraq and Thailand [67].

In the U.S., task shifting may represent a needed strategy for addressing the needs of diverse groups where mental health and social services are typically implemented by degreed professionals. However, in highly diverse areas even well-staffed mental health and social service systems have difficulty providing linguistically and culturally concordant services in native languages. Task shifting can be used in these instances. An example of this is Family Spirit, an early intervention model focused on increasing parenting knowledge as well as maternal and child behavioral outcomes developed specifically for American Indian populations implemented using task shifting with paraprofessional community members who are trained to deliver culturally competent services [66].

Task shifting may be a particularly important approach to employ in refugee resettlement communities [28]. Shifting service delivery to trusted community members can eliminate the most instransigent barriers (language, culture, lack of trust), but the approach poses its own challenges including quality concerns, safety concerns (depending on the tasks), and professional and institutional resistance [42].

Emerging research is beginning to document key components that are necessary to ensure the successful implementation of a task shifting approach. For instance, Deller and colleagues [36] noted that task shifting training should include competence-based education, certification processes, and clear performance standards for those workers who are being “shifted to.” Further, supervision approaches that can ensure the maintenance of quality of services provided by non-professionals is imperative and should include assessment and supportive supervision [18, 68]. These recommendations are commensurate with best practices in the implementation of behavioral parenting programs which typically include competence-based training with certification and field support. Thus, many of the implementation requirements to support a successful task shifting approach for such programs may already exist.

### Study aims

There are two primary aims of the study. The first is focused on curriculum adaptations to create culturally appropriate content of a behaviorally-based parenting model, SafeCare©, to be implemented in a community of refugees and immigrants. An adaptation team consisting of model experts, community members, and service providers will adapt SafeCare for Afghan, Burmese, and the Congolese communities. The second aim is focused on an exploration of task shifting by testing two approaches to a community-based SafeCare implementation: (1) implementation as usual via delivery by providers at community-based agencies and (2) a task-shifted approach in which services are delivered by citizens of the community who do not necessarily possess professional backgrounds and education or experience. As previously mentioned, the traditional SafeCare implementation approach is commensurate with Deller’s [36] recommendations for key components pertaining to the training and support of a successful task shifting implementation. Thus, the primary difference in this aim will be about *who* is delivering the program. We will examine implementation metrics including clients served, model fidelity, adaptations, client skill acquisition, and cultural acceptability. We will also examine family outcomes for families served in each method including parenting behavior, parent-child relationship, parenting stress, and child social and emotional health.

## Method

### Setting

The project will take place in Clarkston, Georgia. The city is just over one square mile in size, but is host to one of the most diverse populations of any municipality in the country, and has been referred to as “the most diverse square mile in America” [79]. Between 2008 and 2012, nearly 75,000 refugees arrived in the U.S., and 12,164 of those refugees arrived in DeKalb County, where Clarkston is located. These refugees hail from over 75 different countries, represent about 150 different ethnic groups, and speak over 60 different languages.

The refugees resettling in Clarkston face severe challenges once they arrive in U.S. In Clarkston, the unemployment rate is 10.9%, the uninsured rate is 31.5, and 43.9% of the population lives beneath the poverty line. Families resettling in Clarkston face difficulties across numerous domains that limit participation in society and optimal health, including access to transportation, affordable housing, healthcare, education, employment, communication across language barriers, immigration policies, and involvement in the criminal justice system. Regarding physical health issues, nearly 50% of arriving refugees have dental problems of some kind, over 20% test positive for tuberculosis, and nearly 20% carry some form of parasite. Refugees are screened only briefly for mental health issues, and given the context of their migration and the clinical manifestations commonly seen in the community safety net clinics, resettlement agencies, and social service providers, it is reasonable to assume that the rates of PTSD, depression, anxiety, and a host of other psychiatric challenges are also very high.

### Aim 1

The goal of Aim is to create a culturally and linguistically competent adaptation of the parent-child interaction portion of the SafeCare model.

#### The SafeCare model

SafeCare is a behavioral parenting program that was developed in 1979, and formal dissemination of SafeCare began in 2007 when the National SafeCare Training and Research Center (NSTRC) was formed. SafeCare addresses three parenting skills critical for the promotion of positive parenting skills and the prevention of child maltreatment among parents of children ages 0–5: parent-child interactions, home safety, and child health. A substantial body of research supports SafeCare including development and validation research, field evaluation, and rigorous randomized trials. The protocols for SafeCare were developed and validated with multiple single-case studies for parent-child interaction [29, 32, 56], home safety [6, 61, 89], and health [12, 35]. Initial uncontrolled group trials of SafeCare [45, 76] demonstrated very large and clinically significant changes in the targeted parenting skills, and quasi-experimental evaluations of SafeCare suggested it reduced child maltreatment recidivism compared to a control group [44, 57]. Recent rigorous randomized trials of SafeCare have shown positive results on both child maltreatment recidivism [24] and parenting skills [19, 93]. Virtually all studies of SafeCare have been conducted in field settings where the effectiveness of transported evidence-based models has historically been attenuated [31]. The model also has been well received by consumers and seen as culturally relevant by diverse groups including Latino and American Indian populations [33, 34].

Our study will focus on the *parent-child interaction module* (PCI) of SafeCare because it most directly addresses the type of positive, nurturing behaviors that have been demonstrated to promote positive parenting and improved children’s development outcomes and is largely a preventive intervention. The PCI module includes two separate protocols, one for parents of infants 0–12 months, and one for parents of children 1 to 5 years old. Both protocols promote positive parent-child relationships by teaching parents to attend to their children and interact with them through language, stimulating play activities, and positive verbalizations and touch. Parents of toddlers and older children are also taught to structure activities using planned activities training [78] in which parents are taught to talk with their child about activities, engage them in making choices, explain rules, provide positive reinforcers while ignoring minor misbehaviors, and give positive consequences for the child’s success [55]. Data from studies focusing only on the PCI module of SafeCare, including a randomized trial, indicates that it is effective at changing parent behavior [29, 32, 56], and child outcomes [19, 53].

#### Prior adaptations to SafeCare

SafeCare has been broadly implemented in the U.S. (30 states), and in several non-U.S. countries (e.g., U. K, Spain, Israel, Australia, Canada), but non-U.S. implementations have primarily been conducted in Western societies. Recently, through NIH funding, an implementation of an adapted version of SafeCare has begun in Kenya to test feasibility [80]. In many of the implementations adaptations have been made, but these are typically fairly minor and include changing language, adding content that is specific to a particular culture, and altering activities such as play activities to fit within a cultural context. In the adaptations made to date, little of the primary content of the SafeCare model has been altered; most changes have been surface adaptations.

#### Creation of adaptation team

Following Aarons and colleagues [1] process for designing adaptations, we created an adaptation team that consisted of model experts (i.e., training staff from the SafeCare training center), individuals with expertise in service delivery (staff from implementing agencies), and community members with cultural expertise (community health workers hired to deliver SafeCare). The goals of the adaptation team were to examine the SafeCare curriculum and the implementation and delivery process, create modifications that are linguistically and culturally relevant to the targeted populations, and to plan implementation adaptations. The team would create initial adaptations but would also address emerging issues during implementation requiring adaptation (e.g., client driven issues, engagement strategies, provider knowledge and skill, resource availability, etc.).

We used Barrera and Castro’s [7] framework for cultural adaptation which includes four basic stages: (1) information gathering, (2) preliminary adaptation design, (3) preliminary adaptation test, (4) adaptation refinement. The focus in the first year of the project will be on stages 1 and 2, information gathering and preliminary adaptation design, and as implementation begins, the focus will shift to stages 3 and 4.

The first stage of *information gathering* consists of activities designed to understand the form and content of needed adaptations and to understand whether the intervention addresses the root causes of the problem in the targeted population. In this phase, the team will discuss the basic concepts of intervention – positive parenting and the specific foci of the SafeCare modules – and will gather data on the relevance and importance of the topic to the targeted population. We will also discuss the basic intervention strategies used in SafeCare and gather information on how those strategies fit within the targeted cultures (discussed in detail below). Finally, we will discuss the SafeCare delivery process to understand *how* to implement the program most effectively with these particular populations.

In the second stage of the preliminary adaptation, we will deploy the information gathered in the first stage to create an initial adaptation of the curriculum. To do this, we will revise parenting materials and training materials as needed including professional translation into the appropriate languages. Community members will review the professional translations of parent materials into the appropriate language. The third and fourth stages – the *preliminary adaptation test* and *adaptation refinement* – will begin once we initiate SafeCare delivery. Upon delivery, the adaptation team will meet regularly to discuss client reactions to the intervention, and other issues that have arisen that may warrant further adaptation.

#### Adaptation targets

There are two main areas to be considered for adaptation: the curriculum itself and the delivery process. Based on our prior experiences we believe the following areas will warrant attention (note that these are not the only areas to be considered).

- *Basic concepts around child development and SafeCare strategies.* When implementing SafeCare in Belarus, we realized that many service providers did not understand basic concepts forming the basis of SafeCare such as the fact that positive and negative parenting impacts children’s development, including brain development, and that parents could be taught new skills.
- *Reinforcers.* What is the usual role of praise as a reinforcer? What other reinforcers are commonly used? Are there gender or generational differences in the way in which reinforcers are used?
- *Use of language*. How comfortable are parents with talking to their children?
- *Norms for structure.* How comfortable are parents structuring interactions with children?
- *Norms for play.* Is parent-child play normative? How do parents typically play with children? What activities to they engage in?
- *Caregiver roles.* SafeCare is designed to be delivered to the primary caregiver, who is typically a parent. However, in some cultures, caregiving may be more communal, and the identification of a specific person as primary caregiver may be less clear.

Potential adaptations to implementation include the following.

- *Primary intervention targets.* Typically, SafeCare is delivered to the primary caregiver, but we will seek to understand who would serve as the best receiver of SafeCare. It may also be the case that more than one adult in the home will receive the training depending on the norms for each ethnic group.
- *Delivery venue.* Some communities are wary of allowing service providers into their home. This issue will be explored and the delivery context will be adapted to fit the needs of the community members.
- *Individual delivery* versus *group delivery.* SafeCare is typically delivered to an individual in the home to addresses logistical barriers and to utilize the natural context to promote skill generalization. However, group parenting can offer a supportive environment and can yield positive results as well.^112^ Group SafeCare was recently developed and is being implemented in public housing settings in New York City and could be used here.
- *Coaching.* A key aspect of SafeCare training is in-field coaching where service providers’ delivery of SafeCare is observed to ensure model fidelity (either in-person or via audio recording). The coaching process can be adapted to fit the community member’s comfort level with having additional people in the home, trust of recording sessions, and the delivery of the curriculum in a native language that cannot be understood by the individual conducting the coaching.

#### Data collection to document initial adaptations

Several kinds of data will be collected to document the adaptation process and to assess the effectiveness and inclusiveness of the adaptation process. During the initial stage of adaptation (states 1 and 2), we will collect the following data.

1. *Qualitative interviews with parents on parenting norms.* A series of 24 semi-structured interviews with parents from the targeted groups will be conducted to assess parenting norms and practices that may need to be incorporated into the curriculum modifications. Questions focused on how parents typically spend time with children, enjoyable activities, how the partner and extended family typically interacts with children, how decisions are made in the household, what the rules for behavior are in the household and how they are established and enforced, reward and discipline practices, challenges to raising children in the U.S., and how raising children is different in the U.S. compared to the home country.
2. *Documentation of changes.* Graduate assistants who are not part of the adaptation team will attend all of the adaptation team meetings to take notes and record suggested and agreed upon adaptations.
3. *Initial adaptation needs scale.* Prior to beginning adaptations, the team was provided an overview of the SafeCare intervention. Members of the team who will deliver services (i.e., providers from organizations and community health workers) will be asked to complete a nine-item survey to assess their perceptions regarding the need for adaptations. The scale was developed by Finley and colleagues [41] and modified slightly here to assess the need for adaptations, fit of the intervention for their client, mismatches between elements of SafeCare and clients, and the need to adapt the delivery methods (e.g., by adding other interventions, changing the setting, etc.).
4. *Inclusion survey after each meeting.* After each meeting of the adaptation team, each member of the team will be invited to complete a six-item survey to assess strengths and challenges of the meeting, feelings of comfort during the meeting, overall effectiveness of the meeting, as well as any changes they would like to see in future meetings. The intent of the survey is to gauge the extent to which different types of members of the adaptation team felt heard and included throughout the process.
5. *Qualitative interviews with adaptation team members.* Upon completion of the initial adaptation process, the research team will interview all members of the adaptation team using a semi-structured format. Questions focused on the adapted materials (e.g., in what ways are they more appropriate for the targeted populations) and the adaptation process (e.g., perceptions of inclusion, whether certain perspective were not considered, etc.).
6. *Adaptations made.* We will catalog and describe the adaptations made for each of the targeted communities. This will include adaptations to the curriculum itself and to the implementation process.

#### Adaptation process once implementation begins

Once the curriculum is adapted and implementation begins, the adaptation team will continue to meet to discuss implementation and client-driven adaptations. The initial implementation of SafeCare will be carefully monitored in line with Barrera and Castro’s^111^ third and fourth phases, the *preliminary adaptation test* and *adaptation refinement*. The preliminary adaptation test phase typically involves pilot participants. Here, because we expect adaptations to be ongoing and a key question in the study, we will not consider the initial participants pilot participants, but will simply monitor implementation closely as the first participants are enrolled. The goal of the third and fourth phases are to carefully gauge participant reactions to the intervention, discuss any major problems, and make refinements as needed. The adaptation team will continue to meet on a regular basis (biweekly or monthly) depending on the frequency of sessions. Meetings will be coordinated by NSTRC training staff, and as recommended by Barrera and Castro [7] decisions for further adaptations will be made by the team of model experts and cultural experts. If further protocol modifications are needed, we will convene trainees for additional training session.

#### Analyses

Analyses of data for Aim 1 will be primarily descriptive with the goal of understanding what adaptations were made and to evaluate the process. We will document and categorize adaptations made to the curriculum and implementation process. To assess the process, we will compute means and standard deviations of the quantitative measures collected (initial adaptation needs and the inclusion survey) and conduct qualitative coding of adaptation team members perceptions of the process.

### Aim 2

Aim 2 of this project is to examine SafeCare implementation using two different methods: standard implementation (SI) by community-based agencies and task-shifted (TS) implementation in which community members are trained deliver SafeCare directly to families. The study design is a quasi-experimental design in which we will examine both implementation metrics and family outcomes across the two implementation types.

#### Standard implementation

Standard implementation will be carried out by three refugee resettlement agencies Each of these agencies is well-established and has operated in Clarkston for several years. Services typically offered as part of resettlement include: welcoming newly arrived refugees at the airport, assistance with housing and food, medical care, legal assistance, employment, language and cultural integration, and enrolling children in school. While these agencies are focused on supporting new arrivals to self-sufficiency within the first 6 month, they also offer ongoing services to the refugee and immigrant community in Clarkston to promote ongoing adjustment and self-sufficiency.

Standard implementation of SafeCare-PCI at the three refugee resettlement agencies will be conducted by current employees of those agencies who will be trained to deliver SafeCare-PCI. Agencies will receive funding from the project to deliver services, and they must determine who is best suited for carrying out these activities and how other tasks are to be managed to allow for time to deliver the new service. The same agency staff will be part of the adaptation team described above. Agencies will also determine how to ‘package’ services to clients which may affect implementation of the newly adopted service. By packaging, we mean when to offer it, what to offer with it, and whether the new service replaces any existing services.

#### Task-shifted implementation

Task-shifted implementation will be conducted by community health workers (CHWs). CHWs are individuals who are citizens of the Clarkston community and members of the ethnic groups being served and will be hired directly to be part of the project and paid for recruitment and delivery of SafeCare-PCI. As noted above, these CHWs will serve as part of the adaptation team. CHWs are expected to provide their own transportation to sessions.

#### Training and support in delivering SafeCare

All providers will undergo training to deliver SafeCare and will be trained together by the NSTRC using its standard training practices [81, 93]. Training in the full SafeCare model is typically 4 days of face-to-face workshop followed by extensive individual coaching with group-based team support. Coaching typically consists of in-person monitoring or a review of recorded sessions scored for fidelity and followed by feedback to the provider. Team meetings are typically held bi-weekly or monthly depending on the team need. Issues discussed during team meetings include general implementation issues including referrals and recruitment, client fit with the intervention, general response to the intervention, adaptations needed, and other needed services.

Because this study included only one of the SafeCare modules, training is planned for 2 days of workshop with follow up coaching. The coaching process may need to be adapted depending on how implementation is conducted. For example, review of recordings will not be possible if services are delivered in the family’s native language (as we expect it will). In such a case, the coaching process will be adapted; for example, it may include self-assessments, coaches listening to sessions with an interpreter, providers delivering a session in English to an English-speaking family. We will work with the team to develop the most feasible and rigorous process possible.

Monthly team meetings will be held with each of the three implementing agencies and with the CHW’s together. The goal of these meetings is to build cohesion among the team and discuss implementation issues external to the individual delivery of SafeCare. These issues include referrals and client flow, when clients are not appropriate for SafeCare, referrals for concomitant problems families experience, data collection, and any other issues raised by service providers.

#### Referral sources

Referral sources may vary for standard and task-shifted implementation. Recruitment of clients at standard implementation sites will be done primarily from the pool of existing clients already served by those agencies, and referrals received for other services. The resettlement agencies have a range of existing programs and services they offer to families, and families with children in the target age range already being served will be offered SafeCare services. Additional referrals may come from governmental organizations, social service organizations, healthcare providers, and ethnic group organizations.

Recruitment and referrals for services to be delivered by CHWs will be facilitated by the administrative structure of the project. That is, project staff will make connections with community-based referral agencies, advertise the study and services with the help of project stakeholders and community leaders, and direct interested participants to the CHW’s for the service. CHWs are expected to have strong connections to community organizations, social service and health agencies, childcare providers, and ethnic organizations, and those connections should facilitate service referrals. All recruitment efforts will describe the intervention service as a program designed “to build a positive relationship with your child and promote healthy development to be ready for school.”

#### Participant enrollment process

Clients who express an interest in the project will first be referred to the study coordinator for enrollment. Because of language barriers, interested participants will be instructed to call a number, leave their name and contact information, and a member of the research team who speaks the native language will return their call. A research team member will contact the interested participant and describe the full study participation, confirm eligibility and, if eligible, consent the participant and conduct the first assessment. Eligibility criteria include being over 18, being a primary caregiver of a child ages birth through five, and identifying and speaking the language of one of the targeted ethnic/national groups.

#### Assessment of implementation outcomes

We conceptualize this study as primarily an implementation study but are collecting both implementation data and family outcome data because of the unique population involved. Several metrics related to implementation will be collected: service delivery*,* provider fidelity, client skill uptake, client engagement/completion, client satisfaction/relevance, and adaptations.

- *Service delivery*. Service delivery will be measured as a simple count of the number of clients served. This metric is an important indicator of the success of the implementation. The target goal for each of standard implementation and task-shifted implementation is 120 clients over 3 years of implementation (240 total clients served). Success or failure at serving clients may suggest the implementation method is viable or not.
- *Provider fidelity.* Fidelity will be monitored on an ongoing basis, and we expect to collect fidelity metrics monthly for the life of the study (3 years).
- *Client skill uptake.* As a part of SafeCare-PCI, providers observe parents interacting with children in play and routine daily activities and rate their use of the desired skills on a 3-point scale (no use, some use, proficient use).
- *Client engagement/completion.* Client engagement and completion will be a count of the number of sessions completed and whether each family completed the program (all 6 sessions). Note that clients will receive a $25 incentive for completing all six sessions of the intervention.
- *Client satisfaction.* Satisfaction will be measured via a 10-item survey given to parents at the end of the program, and a short questionnaire assessing cultural relevance of the program. Finally, brief qualitative interviews will be conducted with a subset of families (20 per year, total *n* = 60) regarding the program to determine program satisfaction, helpfulness, and effectiveness.
- *Adaptations.* We will document planned and ad-hoc adaptations to both the curriculum and to the implementation process for each of the standard implementation and the task shifted implementation as implementation rolls out. Adaptations will be tracked by ethnic group and will be coded and classified conceptually for reporting purposes.

#### Assessment of family outcomes

Data will be collected from clients at three assessment points: baseline (prior to any intervention), post-SafeCare (around 6–8 weeks after baseline), and six-months after baseline. Assessments will be interviews conducted by research team members who speak the native language of the participants. Participants will receive a $25 incentive for completing each assessment. Measures to be collecting during the assessment are as follows:

- Demographics – standard demographics including current work and living situation, and an assessment of migration path.
- *Positive Parenting Behaviors* will be assessed using the Parenting Young Children Survey, [59] a 21-item scale assessing three dimensions of parenting behaviors: limit setting, proactive positive parenting, and supporting positive behavior.
- *Parent-child Relationship and Child Behavioral Health* will be assessed using the attachment subscale of the Devereaux Early Child Assessment [52].
- *Child health status* will be assessed with the 14-item Functional Status II scale [77].
- *Parenting Stress* will be assessed via the Parenting Stress Index– short form [3], a 36-item scale designed to measure stressors in parenthood including parental distress, dysfunctional interactions, and stressors related to having a difficult child.
- *Parent trauma and trauma symptoms* will be assessed with the Harvard Trauma Questionnaire [64] a 17-item scale used to assess both experiences of trauma and trauma symptoms that has been extensively validated in refugee and international populations.
- *Parent mental health* will be assessed with the Hopkins Symptom Checklist [37], a 25-item scale assessing depression, anxiety, and somatic symptoms.
- *Social support* will be assessed with 10 items from the Protective Factors Survey [30] which includes concrete and emotional support subscales.
- *Family Resources* – the 40-item Family Resources Scale – Revised [91] assesses the adequacy of family needs in four domains: basic needs, money, time for self, time for family.
- *Post-migratory problems –* The Post Migration Living Difficulties Scale (PMLD [84];) is a scale on which respondents rate 24 stressors on the extent to which the issue has caused them problems.
- *Parent health status and quality of life* will be assessed with the World Health Organization, Quality of Life Scale [95] a 26-items scale assessing physical and mental health, and overall quality of life.

#### Analyses

Analyses will focus on understanding implementation and outcome differences between standard and task shifted implementations.

##### Implementation metrics

For the implementation metrics, some data exist at the provider level (families served, fidelity), while others exist at the family level (completion of the program, skill acquisition, satisfaction, perceived cultural relevance). Provider level implementation metrics, including fidelity and adaptations, will be analyzed descriptively because of too few observations for statistical analyses. We will examine mean fidelity and patterns of fidelity over time as adaptations are made. For adaptations, we will describe qualitatively the nature of the adaptations and classify those adaptations conceptually. We will also track adaptations made by SI vs. TS providers by ethnic group. For implementation metrics that exist at the family level (program completion, program satisfaction), we will have either one measurement point (completion of SafeCare, consumer satisfaction, cultural relevance) or two (skill acquisition). For metrics with one measurement time point, analyses of differences between implementation groups will be a simple t-test for continuous variables (satisfaction, cultural relevance) or chi-square analyses for categorical variables (completion). If covariates are needed in the models because of baseline differences, a regression framework will be adopted using linear regression for continuous outcomes and logistic regression for dichotomous outcomes. For skill acquisition, which has two measurement time points, a regression framework will be adopted in which the Time 2 skills are regressed onto Time 1 skills, implementation group, and any relevant covariates.

##### Family outcomes

For family outcomes, we will collect data from 240 families (120 SI and 120 TS) at three time points: baseline, post-intervention and four-month follow up (6 months after baseline). With three data points over time, family outcomes will be analyzed in a multilevel context via a GLMM framework with Time nested within Family. The goal is to examine differences in family outcomes between families served by standard and task-shifted implementation, and thus the primary independent variable will be implementation group. The primary outcomes are parent-skills, the parent-child relationship, and parenting-stress. The sample size of 240 was determined via a power analysis and plans to examine differences by implementation type. The sample size provides sufficient power to detect small-to-medium sized differences (*d* = .35–.45) between groups depending on the client attrition (up to 20%) and number of time points.

## Discussion

The goal of this study is to adapt, implement, and evaluate the efficacy of an evidenced-based parenting program (SafeCare) in three ethnic groups in a highly ethnically diverse community. The study will employ the dynamic adaptation processed outline by Aarons and colleagues [1] and use Barrera and Castro’s [7] framework for cultural adaptation to adapt the SafeCare-PCI curriculum for the Afghan, Burmese, and Congolese communities in Clarkston, GA. We will then test the implementation of the adapted curriculum using standard implementation methods (community-based organizations) and a task-shifted method in which implementation is conducted by health workers who are citizens of the community. Both implementation and outcome metrics will be collected.

### Innovation

There are several innovative aspects to this project. First, though standard evidence-based practices such as SafeCare are adapted regularly for disparate populations, this project is simultaneously adapting the curriculum for three groups and delivering it to populations of new Americans. The adaptation process we will engage in is unique in that it will be conducted by a team consisting of model experts, service providers, and community members who will create a culturally relevant curriculum and delivery process. Typically, adaptations to programs are done with a single ethnic group; here we will simultaneously adapt the intervention protocol for three ethnic groups. We will document the adaptations that occur in the field based on consumer demand.

A second major innovation is the application of the task shifting approach in this setting. Task shifting, or using a lay workforce, has been successfully implemented in low income countries to address a range of issues including HIV and mental health problems. Task shifting has been used in the U.S. to address workforce shortage issues (e.g., responsibility for prescribing some drugs has been shifted from physicians to nurse-practitioners), but, to our knowledge, it has not been implemented using a workforce of lay professionals trained to implement preventive family-based or mental health services in the U. S with highly diverse populations. This study will allow us to carefully monitor and document the successes, challenges, and barriers of a task-shifted workforce relative to a standard workforce. We will also track family outcomes to determine if differences are seen between implementation by a standard workforce relative to a task-shifted workforce. This will provide new information on (1) whether an evidence-based intervention like SafeCare can be delivered with fidelity and high consumer satisfaction, and (2) whether family outcomes differ for a task-shifted implementation of SafeCare compared to a standard implementation.

A final innovation will be the development of an adaptation process that can be broadly applied to diverse populations. There is a clear need for EBPs to be made culturally and linguistically appropriate both in the US and abroad. The process we will undertake will serve as a model for transporting effective interventions to refugee camps, displaced populations in non-US countries, and even domestic settings where a great deal of diversity exists in the community. This is critically important given the proliferation of global migration [48].

### Limitations

There are several limitations of the current study. First, only one of the three SafeCare modules is being implemented. The decision to implement only one of three modules was driven primarily by the resources needed for adaptation, translation, implementation, and evaluation; doing all three modules simply was not feasible. Second, for Aim 2, we are collecting both implementation and family outcomes; however, there is no true “no intervention” comparison group other than the SafeCare refusers on which to judge the impact of the intervention on family outcomes. The decision to not have a more rigorous comparison group was driven by the fact that this is an implementation study with the primary question to be addressed focused on how to implement interventions like SafeCare in the targeted communities. The effectiveness of such programs may be in question; however, there is a body of research suggesting that both adapted [16, 72] and un-adapted [22, 58] versions of behavioral parenting programs have strong efficacy among racial and ethnic minority. Still, the populations to be served in this study are unique, and the full effectiveness of evidence-based intervention should not be assumed.

## Data Availability

Not applicable.
